# Impact of the Hospital Frailty Risk Score on Outcomes After Transcatheter Aortic Valve Replacement in Late Elderly Patients

**DOI:** 10.7759/cureus.68922

**Published:** 2024-09-08

**Authors:** Chikara Ueki, Eiji Nakatani, Hideaki Kaneda, Hatoko Sasaki

**Affiliations:** 1 Graduate School of Public Health, Shizuoka Graduate University of Public Health, Shizuoka, JPN; 2 Department of Biostatistics and Health Data Science, Graduate School of Medical Science Nagoya City University, Nagoya, JPN; 3 Department of Research, Okinaka Memorial Institute for Medical Research, Tokyo, JPN

**Keywords:** claims database, frailty, hospital frailty risk score, long-term mortality, transcatheter aortic valve replacement

## Abstract

Objectives: Prognostic prediction using objective indices is needed to optimize the indications for transcatheter aortic valve replacement (TAVR). We evaluated the impact of the Hospital Frailty Risk Score (HFRS), an International Classification of Diseases (ICD)-based frailty index, on the prognosis after TAVR in the late elderly.

Methods: We identified patients aged ≥75 years undergoing TAVR from April 2014 to September 2020 from the Shizuoka Kokuho Database (SKDB). Cox logistic regression analysis was performed to examine predictors of long-term mortality. We also evaluated the relationship between HFRS categories (low risk: <5, intermediate risk: 5-15, high risk: >15) and functional decline.

Results: This study involved 607 patients (189 (31.1%) men) with a mean age of 85.0 years. During the median follow-up period of 20 months, survival significantly differed among HFRS categories (survival at two years; low (HFRS <5): 88.9%, intermediate (HFRS 5-15): 82.6%, high (HFRS >15): 67.7%; log-rank p = 0.002). In the multivariate regression model, male sex (hazard ratio (HR): 2.15, 95% confidence interval (CI): 1.42-3.24), preoperative care needs level of ≥3 (HR: 2.43, 95% CI: 1.17-5.06), and HFRS (HR: 1.07, 95% CI: 1.03-1.12) were significant predictors of mortality. Functional decline-free survival significantly differed among HFRS categories (event-free survival at two years; low: 79.4%, intermediate: 75.2%, high: 50.8%; log-rank p = 0.001).

Conclusions: The HFRS is a predictor of long-term mortality after TAVR in the late elderly and is associated with postoperative functional decline. The HFRS can provide additional information for decision-making regarding treatment strategies for the late elderly.

## Introduction

Transcatheter aortic valve replacement (TAVR) is an established treatment for aortic stenosis in high-risk patients, such as the late elderly. However, TAVR is not recommended for patients with a life expectancy of less than one year in the current American College of Cardiology/American Heart Association guideline [1]. Therefore, evaluating the prognosis of patients undergoing TAVR is of great importance. Preoperative frailty is a major risk factor for morbidity and mortality after TAVR. Frailty assessment is thus recommended in current guidelines to optimize patient selection for TAVR [1, 2]. Although frailty is often assessed using a subjective approach in clinical settings [3], evaluation using a combination of different objective estimates is recommended [2].

Several studies have been performed to evaluate preoperative frailty using frailty indices based on claims data [4-6]. The Hospital Frailty Risk Score (HFRS) based on the International Statistical Classification of Diseases and Related Health Problems, Tenth Revision (ICD-10) [7] was recently developed to predict in-hospital adverse outcomes in patients aged ≥75 years [8] and can reportedly help predict long-term outcomes [9]. Based on the clinical question of whether the HFRS can be used to predict the prognosis of patients undergoing TAVR, several studies have revealed an association between the HFRS and clinical outcomes after TAVR [10-12]. However, no reports have addressed the impact of the HFRS on the long-term prognosis beyond one year after TAVR, and little is known about the effect of the score on the functional decline after TAVR.

The present study was performed to investigate the impact of the HFRS on all-cause mortality and functional decline after TAVR in late elderly patients by using claims data.

## Materials and methods

Ethics statement

We anonymized the Shizuoka Kokuho Database (SKDB) data [13]. The ethics committee of the Shizuoka Graduate University of Public Health approved the study protocol (institutional review board (IRB) number #SGUPH_2021_001_015; approval date June 22, 2021).

Database

The SKDB is the first prefecture-wide longitudinal dataset, and it includes the records of more than two million residents of Shizuoka Prefecture, located near the center of Japan [13]. We collected comprehensive individual-linked data from the SKDB and assigned unique identifiers to everyone. The contents of the dataset are basic information from subscriber lists (age, sex, postal code, observation period, and reasons for withdrawal, including death) and claims data of public health insurance agencies (National Health Insurance: age of <75 years and Latter-Stage Elderly Medical Care System (LSEMCS): age of ≥75 years).

The SKDB is an appropriate database for real-world studies because it includes precise information about death and loss to follow up on in its basic resident registration system. In Japan, residents aged ≥75 years should be enrolled in the LSEMCS. Therefore, the SKDB includes a large portion of residents aged ≥75 years in Shizuoka Prefecture.

Study design and patient population

We conducted a retrospective population-based cohort study using the SKDB. Patients aged ≥75 years were included in the study if they had at least one procedural code for TAVR (Japanese insurance claim codes 150369950, 150387310, and 150387210, which are identical to the ICD-10 Procedure Coding System code 02RF3xx) from 1 April 2014 to 30 September 2020. All participants subscribed to the LSEMCS within the study period. The follow-up period was defined as withdrawal from the LSEMCS or the end of the study (30 September 2020). The reasons for withdrawal included death, emigration from Shizuoka Prefecture, changes of the insurer, and other reasons.

The HFRS

In the present study, preoperative frailty was defined using the HFRS. The HFRS was developed by Gilbert et al. [8] to assess frailty in patients aged ≥75 years using ICD-10 codes. The HFRS is based on 109 ICD-10 codes [7]. Each ICD-10 code was given a specific weight (0.1-4.4), and a sum of the weights of the ICD-10 codes for each patient was calculated as the HFRS of the patient. In the Japanese claims coding system, only the codes corresponding to 91 of the 109 ICD-10 codes were available. Therefore, in this study, the HFRS was calculated using these 91 ICD-10 codes shown in Table 1.

**Table 1:** ICD-10 codes used to calculate HFRS HFRS: Hospital Frailty Risk Score; ICD-10: International Statistical Classification of Diseases and Related Health Problems, Tenth Revision. The HFRS [8] uses ICD-10 [7] codes to assess frailty in patients aged 75 years and older. Due to limitations in the Japanese coding system, the score was calculated using 91 out of the 109 original ICD-10 codes in this study. Data are presented as numbers (%). Bold type indicates statistical significance.

ICD codes	Points	All patients	Risk category of HFRS			p-value
			Low (n=289, 47.6%)	Intermediate (n=294, 48.4%)	High (n=24, 4.0%)	
G81 Hemiplegia	4.4	2 (0.3)	0 (0.0)	2 (0.7)	0 (0.0)	0.538
G30 Alzheimer’s disease	4.0	26 (4.3)	1 (0.3)	18 (6.1)	7 (29.2)	<0.001
I69 Sequelae of cerebrovascular disease	3.7	91 (15.0)	9 (3.1)	69 (23.5)	13 (54.2)	<0.001
R29 Other symptoms and signs involving nervous and musculoskeletal systems	3.6	3 (0.5)	0 (0.0)	2 (0.7)	1 (4.2)	0.059
N39 Other disorders of urinary system	3.2	7 (1.2)	0 (0.0)	7 (2.4)	0 (0.0)	0.030
S00 Superficial injury of head	3.2	32 (5.3)	1 (0.3)	25 (8.5)	6 (25.0)	<0.001
F05 Delirium	3.2	12 (2.0)	2 (0.7)	8 (2.7)	2 (8.3)	0.023
R31 Unspecified hematuria	3.0	20 (3.3)	3 (1.0)	16 (5.4)	1 (4.2)	0.008
R41 Other symptoms and signs involving cognitive functions and awareness	2.7	2 (0.3)	1 (0.3)	1 (0.3)	0 (0.0)	0.999
I67 Other cerebrovascular diseases	2.6	37 (6.1)	5 (1.7)	26 (8.8)	6 (25.0)	<0.001
R26 Abnormalities of gait and mobility	2.6	16 (2.6)	1 (0.3)	13 (4.4)	2 (8.3)	<0.001
R56 Convulsions	2.6	0 (0.0)	0 (0.0)	0 (0.0)	0 (0.0)	-
R40 Somnolence	2.5	6 (0.9)	0 (0.0)	5 (1.7)	1 (4.2)	0.025
S06 Intracranial injury	2.4	4 (0.7)	0 (0.0)	4 (1.4)	0 (0.0)	0.255
T83 Complications of genitourinary prosthetic devices	2.4	1 (0.2)	1 (0.3)	0 (0.0)	0 (0.0)	0.516
E86 Volume depletion	2.3	84 (13.8)	17 (5.9)	58 (19.7)	9 (37.5)	<0.001
E87 Other disorders of fluid	2.3	76 (12.5)	10 (3.5)	57 (19.4)	9 (37.5)	<0.001
M25 Other joint disorders	2.3	44 (7.3)	9 (3.1)	27 (9.2)	8 (33.3)	<0.001
S42 Fracture of shoulder and upper arm	2.3	5 (0.8)	1 (0.3)	4 (1.4)	0 (0.0)	0.488
R54 Senility	2.2	0 (0.0)	0 (0.0)	0 (0.0)	0 (0.0)	-
F03 Unspecified dementia	2.1	11 (1.8)	2 (0.7)	5 (1.7)	4 (16.7)	<0.001
F01 Vascular dementia	2.0	1 (0.7)	0 (0.0)	1 (0.3)	0 (0.0)	0.999
L03 Cellulitis	2.0	11 (1.8)	1 (0.3)	9 (3.1)	1 (4.2)	0.016
S80 Superficial injury of lower leg	2.0	0 (0.0)	0 (0.0)	0 (0.0)	0 (0.0)	-
E53 Deficiency of other B group vitamins	1.9	16 (2.6)	1 (0.3)	12 (4.1)	3 (12.5)	<0.001
H54 Blindness and low vision	1.9	2 (0.3)	0 (0.0)	2 (0.7)	0 (0.0)	0.538
G20 Parkinson’s disease	1.8	14 (2.3)	4 (1.4)	9 (3.1)	1 (4.2)	0.220
K59 Other functional intestinal disorders	1.8	301 (49.6)	84 (29.1)	195 (66.3)	22 (91.7)	<0.001
N17 Acute renal failure	1.8	6 (0.9)	0 (0.0)	4 (1.4)	2 (8.3)	0.003
R55 Syncope and collapse	1.8	8 (1.3)	0 (0.0)	8 (2.7)	0 (0.0)	0.013
S22 Fracture of rib(s)	1.8	26 (4.3)	1 (0.3)	18 (6.1)	7 (29.2)	<0.001
L89 Decubitus ulcer	1.7	6 (0.9)	0 (0.0)	5 (1.7)	1 (4.2)	0.025
Z22 Carrier of infectious disease	1.7	0 (0.0)	0 (0.0)	0 (0.0)	0 (0.0)	-
A41 Other septicemia	1.6	7 (1.2)	0 (0.0)	6 (2.0)	1 (4.2)	0.010
I95 Hypotension	1.6	10 (1.7)	4 (1.4)	5 (1.7)	1 (4.2)	0.405
K26 Duodenal ulcer	1.6	11 (1.8)	2 (0.7)	7 (2.4)	2 (8.3)	0.031
L97 Ulcer of lower limb	1.6	6 (0.9)	1 (0.3)	4 (1.4)	1 (4.2)	0.117
N19 Unspecified renal failure	1.6	38 (6.3)	6 (2.1)	26 (8.8)	6 (25.0)	<0.001
R44 Other symptoms and signs involving general sensations and perceptions	1.6	0 (0.0)	0 (0.0)	0 (0.0)	0 (0.0)	-
G40 Epilepsy	1.5	7 (1.2)	1 (0.3)	5 (1.7)	1 (4.2)	0.087
J96 Respiratory failure	1.5	64 (10.5)	17 (5.9)	40 (13.6)	7 (29.2)	<0.001
M19 Other arthrosis	1.5	56 (9.2)	14 (4.8)	38 (12.9)	4 (16.7)	<0.001
E16 Other disorders of pancreatic internal secretion	1.4	5 (0.8)	1 (0.3)	3 (1.0)	1 (4.2)	0.170
M81 Osteoporosis without pathological fracture	1.4	231 (38.1)	68 (23.5)	150 (51.0)	13 (54.2)	<0.001
N18 Chronic renal failure	1.4	96 (15.8)	17 (5.9)	71 (24.1)	8 (33.3)	<0.001
R94 Abnormal results of function studies	1.4	4 (0.7)	0 (0.0)	3 (1.0)	1 (4.2)	0.044
S32 Fracture of lumbar spine and pelvis	1.4	33 (5.4)	5 (1.7)	25 (8.5)	3 (12.5)	<0.001
S72 Fracture of femur	1.4	22 (3.6)	8 (2.8)	12 (4.1)	2 (8.3)	0.231
N28 Other disorders of kidney and ureter	1.3	47 (7.7)	13 (4.5)	33 (11.2)	1 (4.2)	0.007
R33 Retention of urine	1.3	8 (1.3)	1 (0.3)	7 (2.4)	0 (0.0)	0.115
R69 Unknown and unspecified causes of morbidity	1.3	0 (0.0)	0 (0.0)	0 (0.0)	0 (0.0)	-
G31 Other degenerative diseases of nervous system	1.2	1 (0.2)	0 (0.0)	1 (0.3)	0 (0.0)	0.999
G45 Transient cerebral ischemic attacks and related syndromes	1.2	13 (2.1)	5 (1.7)	8 (2.7)	0 (0.0)	0.751
R32 Unspecified urinary incontinence	1.2	1 (0.2)	0 (0.0)	1 (0.3)	0 (0.0)	0.999
R45 Symptoms and signs involving emotional state	1.2	0 (0.0)	0 (0.0)	0 (0.0)	0 (0.0)	-
S09 Other and unspecified injuries of head	1.2	5 (0.8)	1 (0.3)	4 (1.4)	0 (0.0)	0.488
A04 Other bacterial intestinal infections	1.1	1 (0.2)	0 (0.0)	1 (0.3)	0 (0.0)	0.999
A09 Diarrhea and gastroenteritis of presumed infectious origin	1.1	56 (9.2)	18 (6.2)	28 (9.5)	10 (41.7)	<0.001
J18 Pneumonia	1.1	45 (7.4)	11 (3.8)	30 (10.2)	4 (16.7)	0.002
M79 Other soft tissue disorders	1.1	65 (10.7)	19 (6.6)	39 (13.3)	7 (29.2)	0.001
S01 Open wound of head	1.1	4 (0.7)	0 (0.0)	3 (1.0)	1 (4.2)	0.044
E55 Vitamin D deficiency	1.0	0 (0.0)	0 (0.0)	0 (0.0)	0 (0.0)	-
J69 Pneumonitis due to solids and liquids	1.0	9 (1.5)	1 (0.3)	7 (2.4)	1 (4.2)	0.043
R02 Gangrene	1.0	2 (0.3)	1 (0.3)	1 (0.3)	0 (0.0)	0.999
R47 Speech disturbances	1.0	1 (0.2)	0 (0.0)	1 (0.3)	0 (0.0)	0.999
Z93 Artificial opening status	1.0	1 (0.2)	0 (0.0)	1 (0.3)	0 (0.0)	0.999
E05 Thyrotoxicosis (hyperthyroidism)	0.9	15 (2.5)	5 (1.7)	10 (3.4)	0 (0.0)	0.419
H91 Other hearing loss	0.9	6 (0.9)	3 (1.0)	3 (1.0)	0 (0.0)	0.999
M41 Scoliosis	0.9	12 (2.0)	5 (1.7)	6 (2.0)	1 (4.2)	0.474
R63 Symptoms and signs concerning food and fluid intake	0.9	18 (3.0)	4 (1.4)	11 (3.7)	3 (12.5)	0.011
I63 Cerebral infarction	0.8	115 (19.0)	42 (14.5)	63 (21.4)	10 (41.7)	0.002
K92 Other diseases of digestive system	0.8	33 (5.4)	5 (1.7)	22 (7.5)	6 (25.0)	<0.001
M80 Osteoporosis with pathological fracture	0.8	7 (1.2)	1 (0.3)	4 (1.4)	2 (8.3)	0.018
R13 Dysphagia	0.8	11 (1.8)	0 (0.0)	9 (3.1)	2 (8.3)	0.001
Z99 Dependence on enabling machines and devices	0.8	0 (0.0)	0 (0.0)	0 (0.0)	0 (0.0)	-
F10 Mental and behavioral disorders due to use of alcohol	0.7	0 (0.0)	0 (0.0)	0 (0.0)	0 (0.0)	-
J22 Unspecified acute lower respiratory infection	0.7	0 (0.0)	0 (0.0)	0 (0.0)	0 (0.0)	-
N20 Calculus of kidney and ureter	0.7	13 (2.1)	4 (1.4)	8 (2.7)	1 (4.2)	0.265
R00 Abnormalities of heartbeat	0.7	35 (5.8)	12 (4.2)	19 (6.5)	4 (16.7)	0.038
R79 Other abnormal findings of blood chemistry	0.6	0 (0.0)	0 (0.0)	0 (0.0)	0 (0.0)	-
F32 Depressive episode	0.5	34 (5.6)	11 (3.8)	22 (7.5)	1 (4.2)	0.146
M48 Spinal stenosis (secondary code only)	0.5	118 (19.4)	34 (11.8)	78 (26.5)	6 (25.0)	<0.001
S51 Open wound of forearm	0.5	1 (0.2)	1 (0.3)	0 (0.0)	0 (0.0)	0.516
Z91 Personal history of risk factors	0.5	0 (0.0)	0 (0.0)	0 (0.0)	0 (0.0)	-
D64 Other anemias	0.4	83 (13.7)	28 (9.7)	48 (16.3)	7 (29.2)	0.005
E83 Disorders of mineral metabolism	0.4	21 (3.5)	4 (1.4)	13 (4.4)	4 (16.7)	0.001
L08 Other local infections of skin and subcutaneous tissue	0.4	13 (2.1)	3 (1.0)	9 (3.1)	1 (4.2)	0.148
M15 Polyarthrosis	0.4	0 (0.0)	0 (0.0)	0 (0.0)	0 (0.0)	-
K52 Other noninfective gastroenteritis and colitis	0.3	14 (2.3)	6 (2.1)	5 (1.7)	3 (12.5)	0.022
R11 Nausea and vomiting	0.3	33 (5.4)	8 (2.8)	21 (7.1)	4 (16.7)	0.004
R50 Fever of unknown origin	0.1	6 (0.9)	3 (1.0)	2 (0.7)	1 (4.2)	0.178

The diagnosis codes for the three months before the index hospitalization were extracted using the Japanese electronic claims codes linked to the corresponding ICD-10 codes. These codes were used to calculate the HFRS and two summary comorbidity measures: the Charlson comorbidity index (based on 17 ICD-10 codes) [14] and the Elixhauser comorbidity index (based on 31 ICD-10 codes) [15]. The HFRS classification was defined as low risk (<5), intermediate risk (5-10), and high risk (>15) in the same manner as the original report of the HFRS7 as well as a more recent report [16].

Study outcomes

The primary outcome of the present study was the time to death of any cause during the follow-up period. We also identified postoperative functional decline, and another outcome was the time to death or functional decline. Functional decline was defined as deterioration of the care needs level to ≥3.

In Japan, the care needs certification in long-term care insurance (LTCI) is based on the following process [17]: A trained local government official evaluates nursing care needs using a questionnaire on the patient’s current physical and mental status and use of medical procedures. Based on the results, a standardized score is calculated for the applicant’s physical and mental condition, and the time required for care (grooming, bathing, eating, toileting, mobility, eating, assistance with instrumental activities of daily living, behavioral problems, rehabilitation, and medical services) is estimated. A care needs level is then assigned based on the total estimated care minutes. Finally, the Nursing Care Needs Certification Board, consisting of physicians, nurses, and other health and social services experts, confirms the assigned care needs level. The care needs level in LTCI is associated with self-reported functioning, disability, and physical performance among older patients receiving home medical care [18]. In general, care needs level 3 is described as a status in which a person has difficulty getting up and walking on their own and needs nursing care for almost all personal care, such as eating and toileting.

Statistical analysis

Continuous variables are reported as mean ± standard deviation and categorical variables are reported as percentages. Patient characteristics and procedural variables were compared among the groups using analysis of variance, or the Kruskal-Wallis test, for continuous variables and the chi-square test, or Fisher’s exact test, for categorical variables. The survival rate and event-free rate were estimated by the Kaplan-Meier method, and the differences were assessed using the log-rank test. After the univariable screening (carrying forward all with p<0.05), subsequent multivariable Cox regression analysis was performed to determine predictors of all-cause mortality. The strength of correlations between variables was evaluated using Spearman correlation coefficients. If two variables showed high correlation coefficients (>0.40), these variables would not be entered into the multivariable model simultaneously. The goodness of fit of the model was evaluated by calculating the Akaike information criterion and the Bayesian information criterion. All reported p-values are two-sided, and p<0.05 was considered statistically significant. The statistical analysis was performed using JMP Pro 16.2.0 (SAS Institute Inc., Cary, NC).

## Results

Characteristics of patients undergoing TAVR

In total, 607 patients aged ≥75 years undergoing TAVR were identified in the SKDB from 1 April 2014 to 30 September 2020. Table 2 shows the patients’ baseline characteristics. Their mean age was 85.0±4.1 years. Most of the patients (n = 519, 85.5%) were female, and 4.0% (n = 24) of patients had preoperative severe functional impairment with a care needs level of ≥3. The mean Charlson comorbidity index score was 4.1±2.4, and the mean Elixhauser comorbidity index score was 11.7±6.7. The mean HFRS was 5.9±4.2, and most patients were defined as low risk (n = 289, 47.6%) or intermediate risk (n = 294, 48.4%) in terms of the HFRS; only 4.0% (n = 24) were defined as high risk.

**Table 2:** Patient characteristics HFRS: Hospital Frailty Risk Score; ICD-10: International Statistical Classification of Diseases and Related Health Problems, Tenth Revision. Data are presented as number (%), mean ± standard deviation, or median (range). Bold type indicates statistical significance. *ICD-10 codes with a ≥1% frequency of occurrence in the overall cohort and contributing ≥2 points to the HFRS are presented. (All ICD-10 codes are presented in Table 1). Bold type indicates statistical significance.

Variables	All patients (N=607)	Risk category of HFRS			p-value
		Low (n=289, 47.6%)	Intermediate (n=294, 48.4%)	High (n=24, 4.0%)	
Age	85.0±4.1	84.9±4.0	85.1±4.3	84.4±3.8	0.640
Male sex	189 (31.1)	96 (33.2)	86 (29.3)	7 (29.2)	0.602
ICD-10 codes*					
G30 Alzheimer’s disease	26 (4.3)	1 (0.3)	18 (6.1)	7 (29.2)	<0.001
I69 Sequelae of cerebrovascular disease	91 (15.0)	9 (3.1)	69 (23.5)	13 (54.2)	<0.001
N39 Other disorders of the urinary system	7 (1.2)	0 (0.0)	7 (2.4)	0 (0.0)	0.030
S00 Superficial injury of the head	32 (5.3)	1 (0.3)	25 (8.5)	6 (25.0)	<0.001
F05 Delirium	12 (2.0)	2 (0.7)	8 (2.7)	2 (8.3)	0.024
R31 Unspecified hematuria	20 (3.3)	3 (1.0)	16 (5.4)	1 (4.2)	0.008
I67 Other cerebrovascular diseases	37 (6.1)	5 (1.7)	26 (8.8)	6 (25.0)	<0.001
R26 Abnormalities of gait and mobility	16 (2.6)	1 (0.3)	13 (4.4)	2 (8.3)	<0.001
E86 Volume depletion	84 (13.8)	17 (5.9)	58 (19.7)	9 (37.5)	<0.001
E87 Other disorders of fluid	76 (12.5)	10 (3.5)	57 (19.4)	9 (37.5)	<0.001
M25 Other joint disorders	44 (7.3)	9 (3.1)	27 (9.2)	8 (33.3)	<0.001
F03 Unspecified dementia	11 (1.8)	2 (0.7)	5 (1.7)	4 (16.7)	<0.001
L03 Cellulitis	11 (1.8)	1 (0.3)	9 (3.1)	1 (4.2)	0.016
HFRS	5.9±4.2	2.5±1.6	8.3±2.6	16.7±2.1	<0.001
Charlson comorbidity index	4.1±2.4	3.2±2.0	4.8±2.5	5.8±2.2	<0.001
Elixhauser comorbidity index	11.7±6.7	9.3±5.5	13.6±6.8	16.6±7.2	<0.001
Care needs level of ≥3	24 (4.0)	8 (2.8)	11 (3.7)	5 (20.8)	0.002
Follow-up duration, months	20 (0–77)	22 (0–77)	17 (0–76)	20 (0–45)	0.024

Approaches of TAVR and perioperative coronary revascularization

Table 3 shows the procedural variables. Most of the patients (n = 534, 88.0%) underwent TAVR with a percutaneous approach, 4.0% of patients (n = 24) underwent TAVR with a transapical approach, and the remaining patients (n = 49, 8.1%) had no data regarding the approach used. Perioperative coronary revascularization was performed in 50 (8.2%) patients, of whom 48 underwent percutaneous coronary intervention and two underwent coronary artery bypass grafting.

**Table 3:** Procedural variables Data are presented as numbers (%). HFRS: Hospital Frailty Risk Score; TAVR: transcatheter aortic valve replacement

Variables	All patients	Risk category of HFRS			p-value
		Low (n = 289, 47.6%)	Intermediate (n = 294, 48.4%)	High (n = 24, 4.0%)	
TAVR approach					0.777
Transfemoral	534 (88.0)	253 (87.5)	259 (88.1)	22 (91.7)	
Transapical	24 (4.0)	10 (3.5)	14 (4.8)	0 (0.0)	
Unknown	49 (8.1)	26 (9.0)	21 (7.1)	2 (8.3)	
Perioperative coronary revascularization	50 (8.2)	26 (9.0)	23 (7.8)	1 (4.2)	0.776

All-cause mortality

The median follow-up period was 20 months (range, 0-77 months). During this period, 94 deaths occurred, and the overall survival rate (± standard error) was 93.5%±1.1% at one year, 85.0%±1.8% at two years, and 77.7%±2.4% at three years.

**Figure 1:**
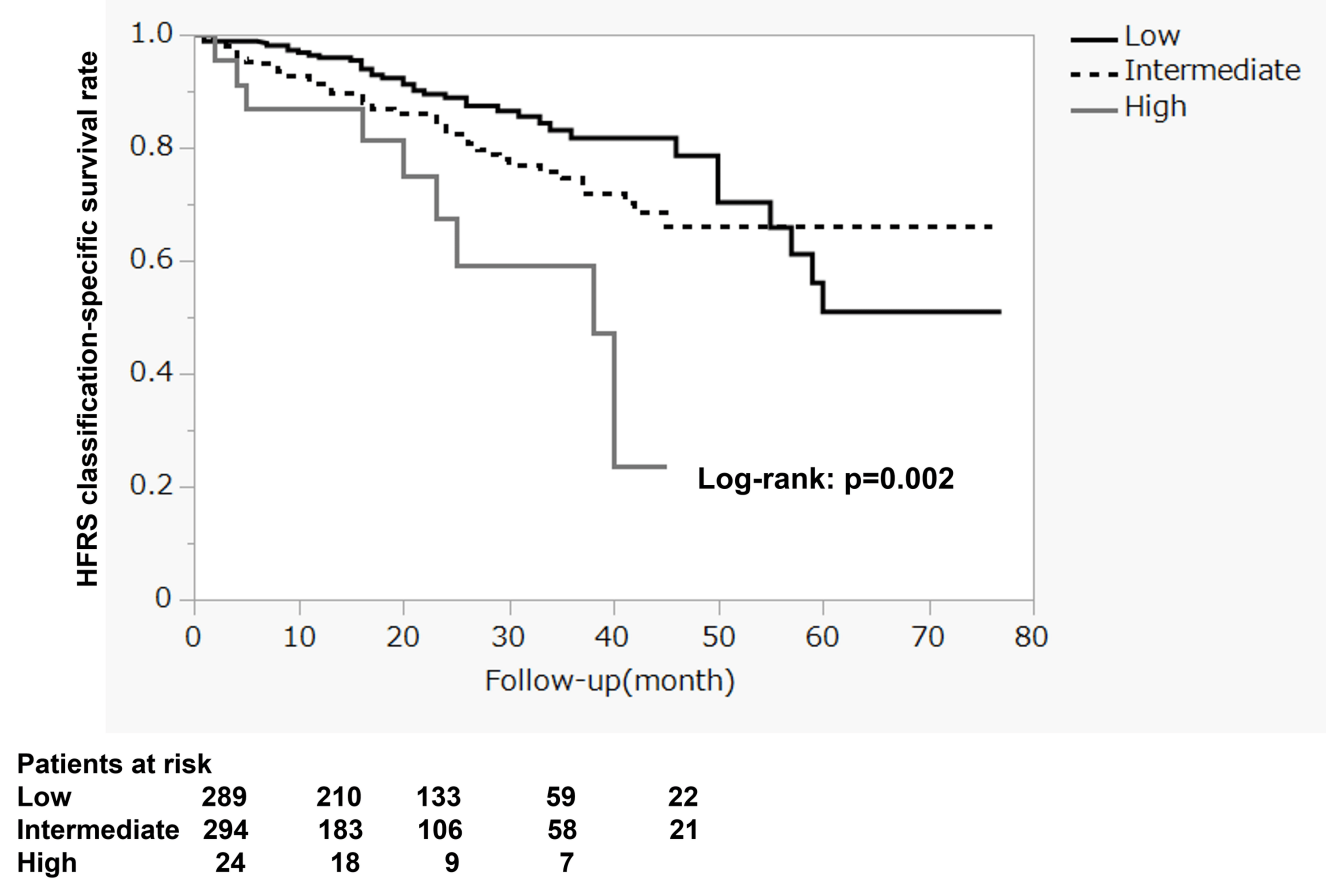
HFRS classification-specific survival rates HFRS: Hospital Frailty Risk Score

The mean one-, two-, and three-year survival rate according to the HFRS classification was 96.0%±1.2%, 88.9%±2.2%, and 81.8%±3.4% in the low-risk group, 91.5%±1.8%, 82.6%±2.8%, and 74.8%±3.7% in the intermediate-risk group, and 87.0%±7.0%, 67.7%±11.4%, and 59.3%±12.7% in the high-risk group, respectively (Figure 1). There were significant differences in the HFRS classification (p = 0.002), and the survival rate decreased as the HFRS category increased in severity.

Prognostic factors for mortality

The results of the univariable screening for predictors of all-cause mortality are shown in Table 4. Significant predictors in the univariable Cox regression analysis were the male sex, preoperative care needs a level of ≥3, the Elixhauser comorbidity index, the Charlson comorbidity index, and the HFRS.

**Table 4:** Univariable Cox regression analysis for all-cause mortality HFRS: Hospital Frailty Risk Score; TA: transapical; TAVR: transcatheter aortic valve replacement; TF: transfemoral Bold type indicates statistical significance.

Variable (reference)	Category or unit	Hazard ratio (95% confidence interval)	p-value
Age	1 year	0.99 (0.94–1.04)	0.732
Sex (female)	Male	2.05 (1.36–3.08)	<0.001
Care needs level (<3)	≥3	2.64 (1.28–5.46)	0.009
Charlson comorbidity index	1 unit	1.12 (1.03–1.21)	0.009
Elixhauser comorbidity index	1 unit	1.04 (1.01–1.07)	0.015
HFRS	1 unit	1.08 (1.03–1.12)	0.002
HFRS (low)	Intermediate	1.48 (0.96–2.27)	0.076
	High	3.53 (1.70–7.35)	<0.001
TAVR approach (TF approach)	TA approach	1.51 (0.61–3.76)	0.375
	Unknown	1.31 (0.75–2.30)	0.344
Perioperative coronary revascularization (absence)	Presence	0.62 (0.25–1.53)	0.265

As a result of evaluating absolute Spearman’s correlation coefficients among these predictors, relatively high correlation coefficients of >0.40 were found between any of the three scores of the Elixhauser and Charlson comorbidity index and the HFRS. The absolute Spearman’s correlation coefficient for the Elixhauser and Charlson index was 0.585, that for the Elixhauser index and the HFRS was 0.419, and that for the Charlson index and the HFRS was 0.443. The correlation coefficients were low (r<0.40) among the other factors. Therefore, we generated three multivariable Cox regression analysis models to prevent multicollinearity between the Elixhauser index, Charlson index, and HFRS.

Table 5 shows the three models of the multivariable Cox regression analysis for predictors of all-cause mortality. Evaluation of the goodness of fit of these models using the Akaike information criterion and Bayesian information criterion revealed that the model, including the HFRS, was the best fit among the three models. In this model, independent predictors of all-cause mortality after TAVR were male sex (hazard ratio (HR), 2.15; 95% confidence interval (CI), 1.42-3.24), preoperative care needs a level of ≥3 (HR, 2.43; 95% CI, 1.17-5.06), and the HFRS (HR, 1.07; 95% CI, 1.03-1.12).

**Table 5:** Multivariable Cox regression analysis for all-cause mortality AIC: Akaike information criterion; BIC: Bayesian information criterion; CI: confidence interval; HFRS: Hospital Frailty Risk Score Bold type indicates statistical significance.

Variable	Model 1		Model 2		Model 3	
	AIC 1033.5, BIC 1046.6		AIC 1033.6, BIC 1046.8		AIC 1027.0, BIC 1040.2	
	Hazard ratio (95% CI)	p-value	Hazard ratio (95% CI)	p-value	Hazard ratio (95% CI)	p-value
Male	1.95 (1.28–2.96)	0.002	1.97 (1.30–2.99)	0.002	2.15 (1.42–3.24)	<0.001
Care needs level of ≥3	2.50 (1.19–5.23)	0.015	2.53 (1.21–5.28)	0.014	2.43 (1.17–5.06)	0.018
Charlson comorbidity index	1.08 (0.99–1.17)	0.083				
Elixhauser comorbidity index			1.03 (0.99–1.06)	0.092		
HFRS					1.07 (1.03–1.12)	0.002

Functional decline in patients without preoperative functional impairment

After excluding 24 patients with preoperative care needs level of ≥3, the remaining 583 patients with preoperative care needs level of <3 were analyzed for postoperative functional decline. In this subgroup, 76 of 583 patients had postoperative functional decline during the follow-up period. The composite outcome of all-cause mortality and functional decline occurred in 136 patients, and the mean functional decline-free survival rate was 88.3%±1.4% at one year, 76.3%±2.1% at two years, and 66.6%±2.8% at three years.

In the univariable Cox regression analysis (Table 6), the significant predictors of all-cause mortality were the Elixhauser comorbidity index (HR, 1.04; 95% CI, 1.01-1.06), the Charlson comorbidity index (HR, 1.09; 95% CI, 1.02-1.17), and the HFRS (HR, 1.05; 95% CI, 1.01-1.09). Because of the relatively high correlation coefficients between any of the three scores, further multivariable Cox regression analysis was not performed.

**Table 6:** Univariable Cox regression analysis for composite outcome HFRS: Hospital Frailty Risk Score; TA: transapical; TAVR: transcatheter aortic valve replacement; TF: transfemoral Bold type indicates statistical significance.

Variable (reference)	Category or unit	Hazard ratio (95% confidence interval)	p-value
Age	1 year	0.99 (0.96–1.04)	0.966
Sex (female)	Male	1.26 (0.56–1.13)	0.207
Charlson comorbidity index	1 unit	1.09 (1.02–1.17)	0.017
Elixhauser comorbidity index	1 unit	1.04 (1.01–1.06)	0.005
HFRS	1 unit	1.05 (1.01–1.09)	0.009
HFRS (low)	Intermediate	1.30 (0.91–1.85)	0.146
	High	3.22 (1.69–6.14)	<0.001
TAVR approach (TF approach)	TA approach	1.00 (0.41–2.46)	0.999
	Unknown	1.24 (0.78–1.99)	0.367
Perioperative coronary revascularization (absence)	Presence	0.62 (0.33–1.37)	0.247

**Figure 2:**
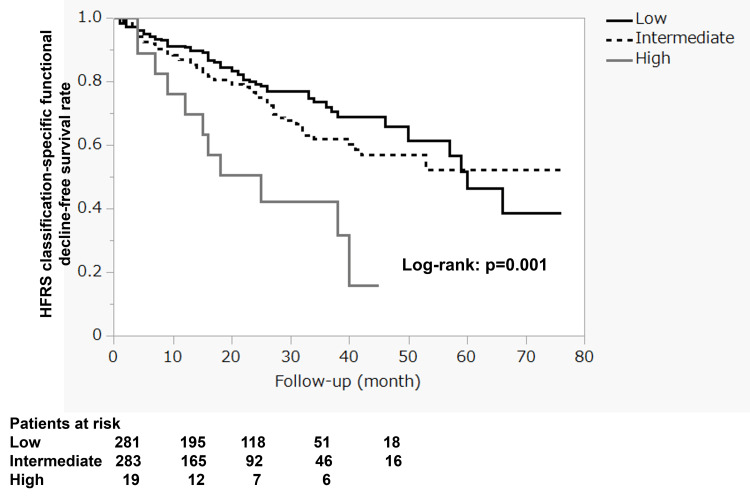
HFRS classification-specific functional decline-free survival rates HFRS: Hospital Frailty Risk Score

When classified by the severity of the preoperative HFRS, the one-, two-, and three-year functional decline-free survival rate was 90.9%±1.8%, 79.4%±2.9%, and 72.2%±3.7% in the low-risk group; 87.0%±2.2%, 75.2%±3.2%, and 62.2%±4.3% in the intermediate-risk group; and 69.8%±11.4%, 50.8%±12.5%, and 42.3%±13.0% in the high-risk group, respectively (Figure 2). There were significant differences in the HFRS classification (p = 0.001), and notably, patients in the high-risk group had a high composite event rate of 30.2% at one year and 49.2% at two years.

## Discussion

In this regional population-based cohort study, we evaluated the impact of the HFRS on all-cause mortality and functional decline after TAVR in late elderly patients. The strengths of this study were the complete coverage of the late elderly undergoing TAVR in the region and the availability of the care needs level extracted from the LTCI data to measure functional impairment. The main findings of this study can be summarized in the following two points. First, the ICD-10-based HFRS was a significant predictor of all-cause mortality, even in the multivariable model including the preoperative care needs level in LTCI, a measure thought to somewhat reflect physical frailty. Second, the high-risk patients stratified by the HFRS showed a very high incidence of mortality and postoperative functional decline (30.2% at one year and 49.2% at two years).

The first point mentioned above is that the HFRS was a significant predictor of midterm mortality in the multivariable model, including the care needs level. Previous studies have shown that frailty is an essential risk factor for adverse outcomes after TAVR [19-21]. Several frailty indices have been developed based on claims data [4-6]. The HFRS, an ICD-10-based frailty score, was recently developed for people aged ≥75 years [8] and has been found to be helpful in predicting short-term mortality and one-year mortality in late elderly patients [9]. However, few reports to date have focused on the predictive impact of the HFRS on the prognosis after TAVR. In a study using the National Inpatient Sample database, Abugroun et al. [11] reported that the HFRS is an independent predictor of mortality, periprocedural complications, and prolonged length of stay among patients aged ≥65 years. Furthermore, Kundi et al. [10] used the nationwide Medicare database to examine the impact of the HFRS on long-term mortality in patients undergoing transcatheter valve therapies. They found the HFRS to be a predictor of one-year mortality after TAVR in patients aged ≥65 years, with a one-year mortality rate of 7.6% in the low-risk group, 17.6% in the intermediate-risk group, and 30.1% in the high-risk group (adjusted HR, 1.06; 95% CI, 1.05-1.07) [10]. The findings of the present study are consistent with these previous studies. However, the present study has two strengths: The first strength is the longer follow-up period (median of 20 months) than in previous studies, which adds evidence regarding the prognostic impact of the HFRS in patients undergoing TAVR. The second strength is the availability of the care needs level, a comprehensive functional status assessment extracted from the LTCI data. In this study, the HFRS was a significant predictor even in the final multivariable model, including the care needs level, which suggests that the HFRS may provide information on high-risk patients not identified by conventional frailty assessments. In other words, the ICD-10-based HFRS and clinical frailty indices may play complementary roles in determining which late elderly patients undergoing TAVR are high-risk.

The second point mentioned above is that the high-risk patients stratified by the HFRS showed a very high incidence (30.2% at one year and 49.2% at two years) of mortality and postoperative functional decline. Additionally, the HFRS was a significant predictor of the composite outcome of mortality and functional decline in the Cox regression analysis (HR, 1.05; 95% CI, 1.01-1.09). Only limited data have been reported on the functional status after TAVR [22]. In an analysis of 106 patients after TAVR, Schoenenberger et al. [23] showed that the frailty index, but not established risk scores, was a predictor of a worsening functional status six months postoperatively. Similarly, in a prospective cohort study of patients undergoing TAVR with 12 months of postoperative follow-up, Kim et al. [24] revealed that patients with severe frailty often showed functional decline and lack of functional improvement after TAVR. However, no studies have been performed to evaluate the association between claims data-based frailty indices, including the HFRS, and functional decline after TAVR. In this study, we defined functional decline as exacerbation of the care needs level using LTCI data and evaluated the association between the HFRS and functional decline after TAVR. Our study revealed a significant difference in the functional decline-free survival rate among the HFRS risk categories, with a meager functional decline-free survival rate in the high-risk HFRS group. Our results are consistent with previous reports [22,23], and patients classified as having severe frailty by the claims data-based indices are also at high risk for postoperative functional decline. Considering that approximately half of patients in the high-risk group die or have difficulty in independent living at 2 years after TAVR, a more conservative decision may be necessary regarding the indication for TAVR for late elderly patients in the high-risk group.

The present study has several limitations. First, because of the nature of claims-based data in the SKDB, this study could not utilize several types of clinical information, including preoperative laboratory data, echocardiographic findings, the type of valves used in TAVR, and postoperative complications. Second, compared with the robust and well-defined data collection in prospective clinical trials or registry studies, ICD diagnostic coding has a potential risk of misclassifying some comorbidities and complications. Third, because our cohort consisted of patients undergoing TAVR after screening by physicians, very high-risk patients were excluded from the cohort in advance. Therefore, as in previous studies, the proportion of patients with a high HFRS (>15) was very low (4.0%), and the absolute number of these patients was also low (n = 24). Given this limitation, further validation of the association between the HFRS and functional impairment after TAVR in a larger patient cohort is warranted. Finally, the relatively small sample size and the specific patient population in this study may introduce potential biases, limiting the generalizability of our findings. Further studies with larger and more diverse cohorts are necessary to confirm these results and to improve the applicability of our findings to a broader patient population.

## Conclusions

The HFRS was a significant predictor of all-cause mortality after TAVR in our model, including sex and preoperative high-care needs level. In particular, patients with a high HRFS (>15) showed high mortality (13.0% at one year, 32.3% at two years, and 40.7% at three years) and an increased incidence of the composite outcome of mortality and functional decline (30.2% at one year, 49.2% at two years, and 57.7% at three years). Considering this low functional decline-free survival rate in the high-risk group, the indications for TAVR in these high-risk patients might be judged more conservatively. Combined with the clinical evaluation of frailty, the HFRS could provide additional information to identify high-risk patients, facilitating appropriate patient selection for TAVR in late elderly patients.
